# Impact of Seasonal Atmospheric Factors and Photoperiod on Floral Biology, Plant–Pollinator Interactions, and Plant Reproduction on *Turnera ulmifolia* L. (Passifloraceae)

**DOI:** 10.3390/biology14010100

**Published:** 2025-01-19

**Authors:** Ujjwal Layek, Nandita Das, Arabinda Samanta, Prakash Karmakar

**Affiliations:** 1 Department of Botany, Rampurhat College, Rampurhat 731224, West Bengal, India; layekujjwal@yahoo.co.in; 2 Centre for Life Sciences, Vidyasagar University, Midnapore 721102, West Bengal, India; nandita.das.mou15@gmail.com; 3 Department of Botany, Jhargram Raj College, Jhargram 721507, West Bengal, India; samanta24bot@gmail.com; 4 Department of Botany & Forestry, Vidyasagar University, Midnapore 721102, West Bengal, India

**Keywords:** flowering intensity, flowering phenology, melittophily, myrmecophily, psychophily, pulsatory pollination

## Abstract

Reproductive traits and plant–pollinator interactions largely depend on seasonal weather conditions, which are species-specific. *Turnera ulmifolia* is an ornamental plant distributed worldwide. There is little information about plant species’ reproductive ecology and environmental factors’ impact on it. Here, we aimed to examine the effects of seasonal atmospheric factors (e.g., temperature, light, relative humidity, rainfall) and photoperiod on flowering, interactions with flower visitors, and the reproductive success of *Turnera ulmifolia* in West Bengal, India. Flowering intensity peaked in hot summers and dropped in cold winters, correlating positively with temperature and humidity. Flower opening and closing occurred earlier on hot days, while flower longevity increased in winter, showing a negative correlation with temperature and humidity. Pollen and ovule production were lower in cold weather, positively linked to temperature and humidity. The self-compatible plant was moderately dependent on pollinators and had no pollination deficit in open conditions. Visitor abundance, richness, and diversity varied season-wise, with higher values during spring–summer. Based on pollinating agents, the plant showed multiple pollination modes (e.g., melittophily, myophily, myrmecophily, and psychophily). Effective pollinators were *Amegilla zonata*, *Borbo cinnara*, *Halictus acrocephalus*, *Nomia* (*Curvinomia*) *strigata*, and *Tetragonula iridipennis*. The fruit set (%) did not differ significantly season-wise, but the seed set remained higher in the hot days of summer than in cold winter. Therefore, it can be concluded that atmospheric factors and photoperiod significantly impact floral traits, plant–pollinator interactions, and plant reproduction.

## Simple Summary

The study depicted the influences of atmospheric factors and photoperiod on floral biology, plant–pollinator interactions, and the reproductive success of *Turnera ulmifolia*. Temperature, light, humidity, and day length enhanced flowering intensity, pollen, and ovule production, while flower longevity decreased with higher temperature, humidity, and longer day length. Visitor traits positively correlated with flowering intensity and were negatively influenced by rainfall. Effective pollinators were *Amegilla zonata*, *Borbo cinnara*, *Halictus acrocephalus*, *Nomia* (*Curvinomia*) *strigata,* and *Tetragonula iridipennis*. They pollinated the flowers via the ventral thorax and abdomen and also through their legs, antennae, proboscis, and wings. *Nomia* (*Curvinomia*) *strigata* also employed pulsatory pollination. The reproductive fitness of the plant species was highest in the hot summer and lowest during the cold winter seasons.

## 1. Introduction

Flowering is an essential plant life cycle phase that strongly affects plant fitness [1,2]. Flowering traits (e.g., flowering phenology and floral morphological and functional traits) are crucial characteristics that determine the reproductive efficacy of plants and are essential for assessing biodiversity threats under changing environmental conditions [3,4,5]. Plants display various flowering patterns (e.g., timing, duration, and frequency) [6,7]. Flowering patterns determine the number of outcrossing, mates, near-neighbour matings, and reproductive output [8,9].

Several aspects of floral biology, including pollen vitality and stigma receptivity, play a crucial role in the reproductive success of a population and are influenced by the timing of flowering [10,11]. The floral traits (including flower longevity, pollen viability, and stigma receptivity) and the overlap periods of male and female functional traits (pollen viability and stigma receptivity) vary across various flowering times and habitats [12,13]. Studies of these floral traits are fundamental prerequisites for understanding the reproductive constraints that affect a given population.

In addition to endogenous genetic components, flowering is determined by various environmental factors, such as day length, temperature, light intensity, moisture, and stress [14,15]. Photoperiod (i.e., day length) is a prime controlling factor for flower induction for many plants [16,17]. The light intensity can affect photosynthesis, phytohormone production, and plant growth [18,19], influencing many plants’ flowering [20,21]. Flowering may be reduced or delayed as irradiance is reduced [22]. Supplementary light irradiance can differentially influence flowering depending on the plant species and light quality. Some researchers (e.g., Armitage and Tsujita [23]) found that supplemental high-pressure sodium (HPS) lighting hastened flowering in some plants compared with plants grown under natural daylight conditions. Air temperature influences flowering through several modes of action. Here, it is essential to distinguish between vernalisation (i.e., the prolonged cold exposure needed for floral transition in many plants) and ambient temperature (i.e., the non-stressful temperature range for a species). In plants, ambient temperature fluctuations impact various physiological and developmental responses―including pigment formation, cell organelle development, net CO_2_ assimilation rates, transpiration, flowering, and seed yield [24,25,26]. Atmospheric humidity also affects floral traits, including anther dehiscence and stigma receptivity [27]. Rainfall significantly affects flowering phenology and floral resource availability for visitors [28,29]. Barrett and Brown [28] showed that absent or delayed rain altered phenological events and resulted in the decreased production of flowers.

Flower-visiting insects are another factor that constitutes one of the most critical interactions for plant reproduction. Plant–pollinator interactions highly depend on floral traits [30,31] and environmental factors [32]. This interaction may drive the evolution of flowering time by influencing competition for pollinators, potentially favouring asynchronous flowering to reduce competition or encouraging synchronous flowering to enhance pollinator efficiency and service quality [1,33]. Moreover, flowering time and synchronisation shifts are linked to the regularity and behavioural patterns of insect visits to flowers, with insect activity patterns typically aligning closely with the flowering phenology of related plant species [2,34]. Changes in the behaviour patterns of flower-visiting insects severely impact plant reproduction [35]. Investigating the relationships among flowering phenology, environmental factors, and insect visitors can offer valuable insights into the selective pressures shaping the evolution of flowering traits.

*Turnera ulmifolia* L. (Passifloraceae) is a polymorphic complex native to the New World tropics, composed of heterostylous and homostylous forms [36]. The majority of heterostylous varieties of *Turnera ulmifolia* are ruderal weeds. In contrast, varieties *angustifolia*, *elegans*, and *intermedia* are also used as garden ornamentals, and man has played a significant role in expanding their ranges. The plant has many pharmaceutical activities, including anti-microbial [37], antioxidant [38], anti-inflammatory [39], and anti-ulcerogenic [39]. However, little information is available about its floral and pollination biology (e.g., Torres-Hernández et al. [40]). In Asian countries, there are no specific records about its reproductive fates. Knowledge of the reproductive biology of a plant species is necessary to manage its population. Therefore, research on floral biology and plant-pollinator interactions on *Turnera ulmifolia* is demanding.

Here, we aimed to provide new information about the reproductive biology of *Turnera ulmifolia* and the impacts of environmental factors on floral biology, plant-pollinator interactions, and plant reproduction. The following aspects were investigated: (a) floral biology, (b) floral visitors and pollinators, and (c) impacts of seasonal atmospheric variables on plant reproduction. We hypothesised that seasonal atmospheric factors and photoperiod might affect the floral biology of *Turnera ulmifolia* and thereby influence the plant-pollinator interactions and reproductive fitness of the plant species.

## 2. Materials and Methods

### 2.1. Study Area and Plant Species

We carried out the present works in Bolpur (23.67° N and 87.72° E) of the Birbhum district, West Bengal, India. Some flower samples were also collected from the Vidyasagar University campus’s surrounding vegetation (Midnapore, Paschim Medinipur, West Bengal; 22.43° N and 87.30° E) for the microscopic study of flower morphological traits. The study area of Bolpur characterises medium-density vegetation [41]. The majority of regions cover human habitats. The study zone exhibits hot summer (April–June, day temperatures at around 35–42 °C), following monsoon (July–August, annual rainfall 1479.9 mm), pleasant autumn (September–mid-October), late autumn (mid-October–mid-November), cold winter (mid-November–January, day temperatures at around 7–15 °C), and spring (February–March).

The present works were conducted on *Turnera ulmifolia* L. var. *angustifolia* (Passifloraceae) during 2022–2023. The plant is native to the New World tropics and distributed worldwide. It is a small shrub that grows erect on roadside walls, cement blocks, and rocks. This homostylous variety is also cultivated as an ornamental.

### 2.2. Environmental Factors

We recorded seasonal atmospheric factors (temperature, light intensity, relative humidity, and rainfall) and photoperiod (i.e., day length) within the study area using handy instruments (thermometer, photometer, and hygrometer, for recording temperature, light intensity, and relative humidity, respectively). Data were taken throughout the day, and average values on a sampling day were considered. On a sampling day, rainfall was recorded as an ordinal number (0—no rainfall, 1—very little rainfall, 2—little rainfall, 3—moderate rainfall, 4—heavy rainfall, and 5—very heavy rainfall).

### 2.3. Floral Biology

Flowering phenological data (flowering frequency, time, flowering pattern) were recorded (according to Gentry [6] and Hopkins [42]). The flowering intensity was measured as the number of freshly opened flowers per day per individual. For this, we selected old, medium, and young individuals (but reached the reproductive phase). We recorded flower opening and closing times on a sampling day (n = 3 flowers per sampling day; 16 sampling days per season). Observations were conducted at one-hour intervals. Then, flower longevity (time between flower opening and closing) was estimated for each season.

Flower morphology was studied with the help of microscopes (compound light microscope: Primo Star, Zeiss; stereo microscope: Stemi 508, Zeiss; scanning electron microscope: Merlin, Zeiss). The methodologies for the SEM study are given in Table S1. All anthers of a flower were taken into a vial before anther dehiscence in the early morning (one flower on a sampling day) to determine pollen grain per flower and preserved in 70% ethanol. During counting pollen grains, anthers were crushed with a glass rod to release the pollen. After shaking, 10 µL of pollen solution was taken on a glass slide, covered with a square-shaped cover slip, and the pollen grains were counted at 10× magnification with a compound light microscope. Then, we estimated the number of pollen grains per flower by considering the total volume of pollen solution. We studied pollen morphology using light microscopy and scanning electron microscopy (see Table S1). Three flowers (one per individual) were randomly chosen on a sampling day (16 sampling days per season) to determine ovule numbers. The ovaries were dissected with a scalpel, placed in a drop of water on glass slides, and slightly pressed to spread out. Ovules were counted under a dissecting magnifying glass.

To assess pollen viability, we used two staining techniques: (i) 2,3,5-triphenyl tetrazolium chloride (TTC) [43] and (ii) iodine potassium iodide (IKI) [44]. A total of 1% TTC (0.1 g TTC and 6 g sucrose dissolved in 10 mL distilled water) was prepared, and a drop of the mixture was taken on a clean glass slide. Pollen grains (fresh pollen collected from a flower during opening, one sample per sampling day) were added to this mixture with a brush and covered with a coverslip. After two hours of incubation, the staining of pollen grains was observed under a compound microscope (counted ≥100 pollen grains). The appearance of a red colour indicated viability, and other pollens (i.e., light red, no change, black, or yellow) were regarded as non-viable. The second staining method dissolved 0.1 g potassium iodide and 0.05 g iodine in 10 mL distilled water for the IKI solution. Pollen grains were added to this solution, and after five minutes of incubation, pollen grains were observed under a compound microscope. Pollen grains stained dark (dark red or brown colour) were considered viable.

Pollen germinability was tested by in vitro germination with an agarose–sucrose medium [45]. We used 10% sucrose, 0.5% agar, and 5 ppm boric acid in the medium. A small amount of medium was taken into a Petri dish, and bulk fresh pollen grains were added to the medium and incubated for 24 h at 25 °C in darkness. Then, pollens with medium were taken on a slide and observed under a compound microscope (counted ≥100 pollen grains). We considered pollen as germinated if the pollen tube length was greater than the diameter of the pollen grains.

Stigma receptivity was checked by using hydrogen peroxide [46]. Fresh flowers (one per time interval of 1 h covering throughout flower longevity) were collected. Stigmas were excised from the flowers using razor blades and placed on a glass slide. Then, a drop of H_2_O_2_ solution was added. After 10–15 s, stigma receptivity was observed under a dissecting microscope. The appearance of many bubbles indicates receptivity. More bubbles indicated stronger stigma receptivity.

### 2.4. Mating System and Plant Reproduction

To determine the mating system, we carried out five pollination treatments: (i) open pollination, (ii) spontaneous auto-pollination, (iii) manual selfing, (iv) manual crossing, and (v) supplementary pollination. We selected flower buds in the late afternoon, and they were marked. We bagged the selected buds (for auto-pollination and manual pollination treatments) with nylon netting until the senescence of floral parts. We utilised 40 flowers for each treatment during summer, except open pollination, which we conducted for each season with 40 flowers. If butterfly larvae damaged selected flowers, we replaced them by selecting new flower buds for that particular treatment. For manual selfing, we uncovered the net at 7.00–8.00 h, added pollen grains to the stigmas of the same flowers, and immediately re-bagged them. Buds selected for cross-pollination were emasculated before anther dehiscence. For the manual crossing, pollen grains were taken from the flowers of different individuals. For supplementary pollination, we manually added pollen grains (from the same and other individuals) to the stigmas in addition to the routine pollination service provided by native pollinators. After 10 days, we recorded each treatment’s fruit and seed sets.

We measured the index of self-incompatibility (ISI) according to Raduski et al. [47] as follows:

$$\text{ISI}=1-\frac{\text{Seed set in self pollination}}{\text{Seed set in cross pollination}}$$

Based on the ISI value, plant species can be placed among the three categories: (i) self-incompatible (ISI ≥ 0.8), (ii) partial self-incompatible (0.2 < ISI < 0.8), and (iii) self-compatible (ISI ≤ 0.2).

We calculated the index of dependency of plants on pollinators (IDP) according to Layek et al. [48] as follows:

IDP=1−Re Rs 

Re is the reproductive success (fruit set or seed set; here, we considered seed set per flower) in pollinator exclusion treatment, and Rs means reproductive success in supplementary pollination treatment. The value of IDP ranges from 0 to 1, and a higher value indicates a higher dependency on pollinators.

To assess whether plant species experience pollen transfer limitations under open field conditions, we calculated the ’coefficient of pollination deficit (D)’ following the approach described by Layek et al. [48]:

$$D=1-\frac{\mathrm{Ro}}{\mathrm{Rs}\;}$$

Ro denotes reproductive success (here, seed set per flower) in open pollination. The value of D ranges from 0 to 1, and a higher value (D ≥ 0.1) indicates a significant pollination deficit.

### 2.5. Floral Visitors

We observed the visitors at four time slots (i.e., 6.00–8.00 h, 8.00–10.00 h, 10.00–12.00 h, and 12.00–14.00 h) covering the flower longevity. Each survey (i.e., plant-based sampling) was continued for 5 min on an individual plant. On a sampling day, we conducted one observation per time slot (n = 4 observations per day; N = 4 × 16 × 6, 16 sampling days per season, 6 seasons). The encountered visitors were identified in the field or captured for later identification.

We estimated the abundance (i.e., the number of individuals of a species/plant/5 min) of each flower-visiting species. For each flower-visiting species, we calculated the relative abundance (RA) as follows (Layek et al. [31]):

$$\mathrm{RA}\;\left(\%\right)=\frac{ni}{N}\times 100$$

The variable n*i* represents the number of individuals recorded for an insect species *i*, while N denotes the total number of individuals recorded across all flower-visiting insect species.

The richness of the flower-visiting community was estimated using the index (D) of Margalef [49] as follows:

$$D=\frac{S-1}{lnN}$$

In this context, S represents the number of flower-visiting species, and N indicates the total number of individuals observed. The natural logarithm is expressed as *ln*. The value of D was calculated for each sample, where one sample corresponds to a single survey (a 5 min observation on an individual plant).

The diversity of flower visitors was determined using the diversity index (*H’*) of Shannon–Weaver [50] as follows:

$$H^{\prime }=-\overset{n}{\underset{i}{\sum }}\left(\mathrm{pi}.ln\mathrm{pi}\right)$$

Here, pi represents the proportion of each visitor species within the sample (pi = n*i*/N, where n*i* is the number of individuals recorded for species *i*, and N is the total number of individuals recorded in the sample).

We recorded the number of visits that received a flower per 5 min duration. Data were taken for all seasons covering all four time slots (N = 2 × 4 × 16 × 6 = 768 observations; n = 2 observations/time slot/sampling day, 16 sampling days per season). We documented the types of floral resources collected by the visitors, including nectar, pollen grains, and floral tissues. The flower visitation rate (VR), or foraging rate, estimated as the number of flowers visited per minute, was recorded with up to 20 observations per insect species. For the visitors with a low visitation rate (e.g., *Tetragonula iridipennis*), we counted the number of flowers visited for 5 min. Then, we converted it for a unit of time (i.e., per minute). The flower handling time (i.e., the duration spent by a visitor on a single flower on a visit) was also measured. For abundant species, N = 20 × 4 = 80 observations were conducted per species across all time slots.

### 2.6. Pollinating Strategies of Visitors

We documented flower visitation events and categorised them as either legitimate or illegitimate. A visit was classified as legitimate if the visitor made contact with the stigmatic surface; otherwise, it was deemed illegitimate. For legitimate visitors, we observed the different modes of pollination: (i) nototribic (pollen deposited on the dorsal side of the visitor), (ii) sternotribic (pollen deposited on the ventral side of the visitor), (iii) noto-sternotribic (pollen deposited on both dorsal and ventral sides of the visitor), and (iv) appendages mediated (i.e., through delicate parts like legs, antennae, proboscis, etc.).

We estimate the single-visit pollination efficiency of some dominant visitors. For this purpose, we bagged matured flower buds in the late afternoon (n = 10 flower buds per observation day; 20 observation days). The next morning, we uncovered the virgin flowers, and after receiving a visit, we re-bagged them immediately and tagged them with the visitor’s specifications. After 10 days, we recorded fruit and seed sets. Then, we estimated the single-visit pollination efficiency index (PE*i*) (according to Spears [51]) of dominant visitors as follows:

$$\mathrm{PE}i=\frac{Pi-Z}{U-Z}$$

P*i* represents the average number of seed sets per flower in the single-visit experiment for the visitor species *i*. Z denotes the average number of seed sets in the pollinator exclusion treatment. U indicates the average number of seed sets resulting from open pollination, where visitation is unrestricted.

We determined a combined parameter, the pollinator importance (PI), for the dominant flower visitors based on the methodology outlined by Layek et al. [52]. We considered the numeric values of relative abundance (RA), visitation rate (VR), and single-visit pollination efficiency index (PE*i*). As flowers are hermaphrodite, the flower sex selection index (FSI) would be 1, and we ignored FSI for the calculation of PI by multiplying the numeric values. Here, PI was calculated as follows:

PI=RA×VR×PEi

A flower visitor species with a high PI value was considered an effective pollinator for the plant species.

### 2.7. Statistical Analysis

The data were analysed descriptively to calculate the mean and standard deviation. The Shapiro–Wilk test was employed to assess the normality of the data distribution. For data that were not normally distributed, the non-parametric Kruskal–Wallis H test was applied (e.g., floral display size, flower longevity, ovule and pollen production, seed set, abundance, richness, diversity of visitors, number of visits per flower, flower visitation rate, and handling time). When the *p*-value was significant (*p* ≤ 0.05), post hoc analyses were conducted using Dunn’s test following the Kruskal–Wallis test. Spearman’s rank correlation coefficient (Spearman’s rho) was used to examine the relationships between environmental factors (e.g., temperature, light, relative humidity, rainfall, and photoperiod) and floral traits, reproductive success, and flower visitors. Statistical analyses were performed using SPSS version 25.0 and R (R Core Team 2022).

## 3. Results

### 3.1. Floral Biology

**Figure 1:**
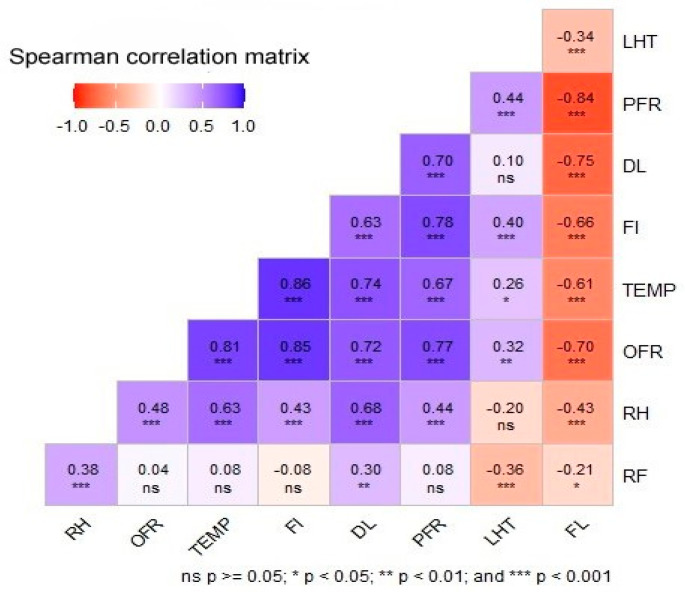
Correlation matrix derived from atmospheric factors and flower traits. DL: day length, FI: flowering intensity, FL: flower longevity, LTH: light intensity, OFR: ovules per flower, PFR: pollens per flower, RH: relative humidity, TEMP: temperature.

*Turnera ulmifolia* bloomed throughout the years and showed a steady-state flowering pattern. All individuals (that reached the reproductive phase) were flowering synchronously. Flowering intensity varied season-wise (Kruskal–Wallis H test: χ^2^ = 52.78, df = 5, *p* < 0.001), with higher during summer–monsoon and lower in winter (Table 1). The flowering intensity was highly positively correlated with temperature (Spearman’s rho = 0.86, *p* < 0.001, n = 96), humidity (Spearman’s rho = 0.43, *p* < 0.001, n = 96), and day length (Spearman’s rho = 0.63, *p* < 0.001, n = 96) (Figure 1). Flower opening time varied season-wise. In warmer seasons (e.g., summer and monsoon), flowers opened earlier (5.00–6.00 h) than in the cold winter (8.00–9.00 h). Flower longevity also varied season-wise (Kruskal–Wallis H test: χ^2^ = 263.52, df = 5, *p* < 0.001). Comparatively, a higher flower longevity was recorded during cold winter (8.25 ± 0.45 h). Flower longevity was negatively correlated with temperature, humidity, and day length.

**Figure 2:**
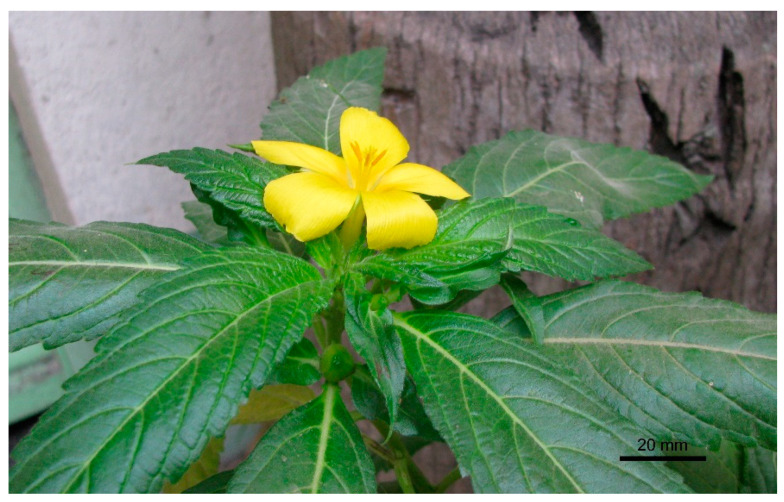
A flowering twig of *Turnera ulmifolia*.

**Figure 3:**
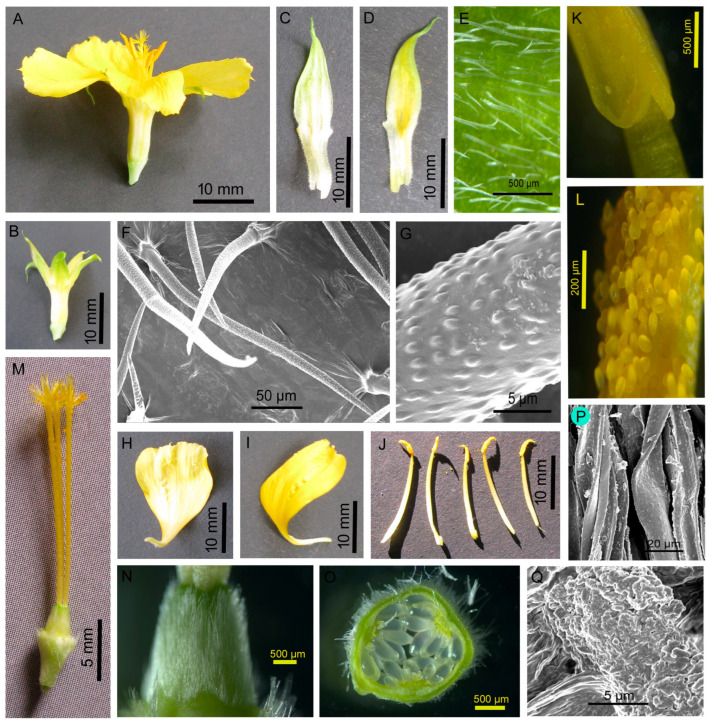
Flower parts. (**A**) Flower, (**B**) calyx, (**C**,**D**) sepal, (**E,F**) a part of sepal showing hairs, (**G**) part of a hair, (**H**,**I**) petal, (**J**) stamens, (**K**) a portion of stamen showing basifixed anther, (**L**) dehisced anther showing pollen grains, (**M**) carpels, (**N**) ovary, (**O**) t.s. of ovary showing placentation, (**P**) hairs of ovary surface, and (**Q**) stigmatic surface.

**Figure 4:**
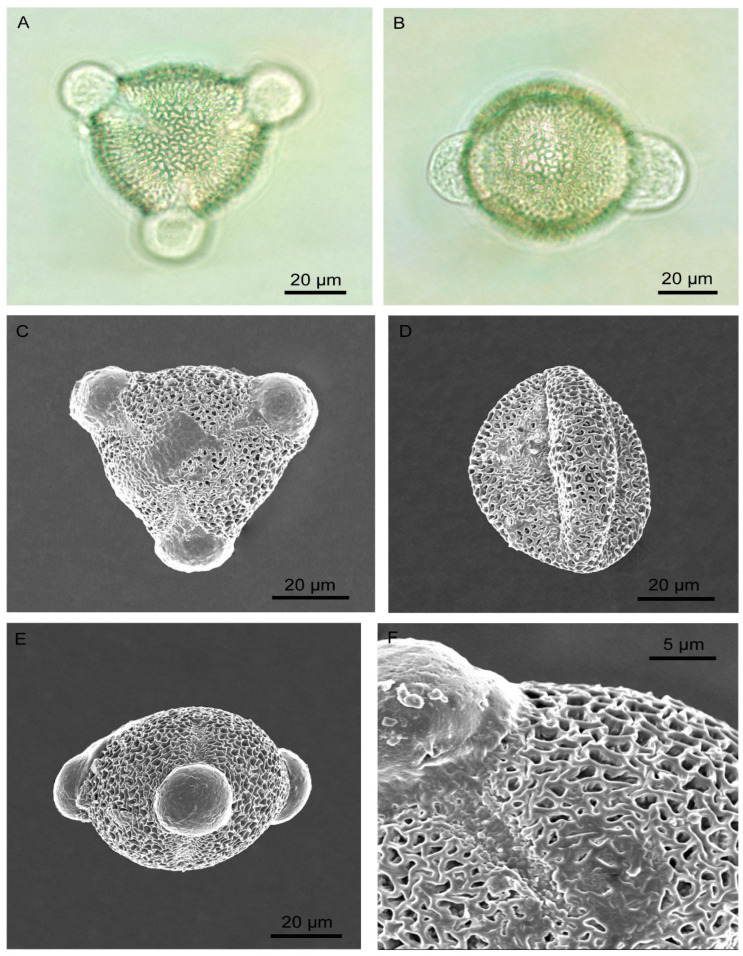
Pollen grains of *Turnera ulmifolia*. (**A**,**B**) light microscopy images, (**A**) polar view, (**B**) equatorial view. (**C**–**F**) scanning electron microscopy images, (**C**) polar view, (**D**,**E**) equatorial view, (**F**) an enlarged view showing exine ornamentations.

Solitary flowers were borne at the tip of a lateral branch (Figure 2). Calyx had five sepals, semi-gamosepalous (partially fused at their lower parts), a light green dorsal surface and light yellow on the ventral side. Each sepal was lanceolate (22.25 ± 1.21 mm long and 5.12 ± 0.67 mm wide), and the outer surface was hairy. The hairs were narrow and elongated (256.53 ± 97.26 µm long and 18.34 ± 2.27 µm wide at the base) and with a beaded surface (Figure 3). The corolla had five petals, polypetalous and yellow. The upper part was broad (18.60 ± 1.50 mm wide), and the base was very narrow. Five stamens were free; the filaments were 15.40 ± 1.19 mm long; anthers were 4.60 ± 0.60 mm, basifixed and dehisced longitudinally. Carpels were three; styles were free, 14.80 ± 0.83 mm long, and light yellow, each terminated with fibrous stigmas. The ovary was dome-shaped, with a greenish and hairy surface. There were many ovules within an ovary, and placentation was the parietal type. The palynomorphic study showed the pollen grains were monad, spheroidal (44.46 µm in diameter), amb triangular, trizonocolporate, exine about 2.5 µm thick, and exine ornamentation was of the reticulate type (Figure 4).

Pollen production (number of pollen grains) per flower was 13,426.68 ± 1008.01 (mean ± SD, n = 96), and ovule per flower was 53.80 ± 11.12 (mean ± SD, n = 288). The number of pollens and ovules varied seasonally (Pollen: χ^2^ = 67.96, df = 5, *p* < 0.001; ovule: χ^2^ = 79.64, df = 5, *p* < 0.001). Pollen and ovule production remained higher during summer–monsoon and lower during winter (Table 1). The ovule-to-pollen ratio was 1:249.57. Pollen and ovule production positively correlated with temperature (pollen: Spearman’s rho = 0.81; ovule: Spearman’s rho = 0.75; *p* < 0.001, n = 96).

**Figure 5:**
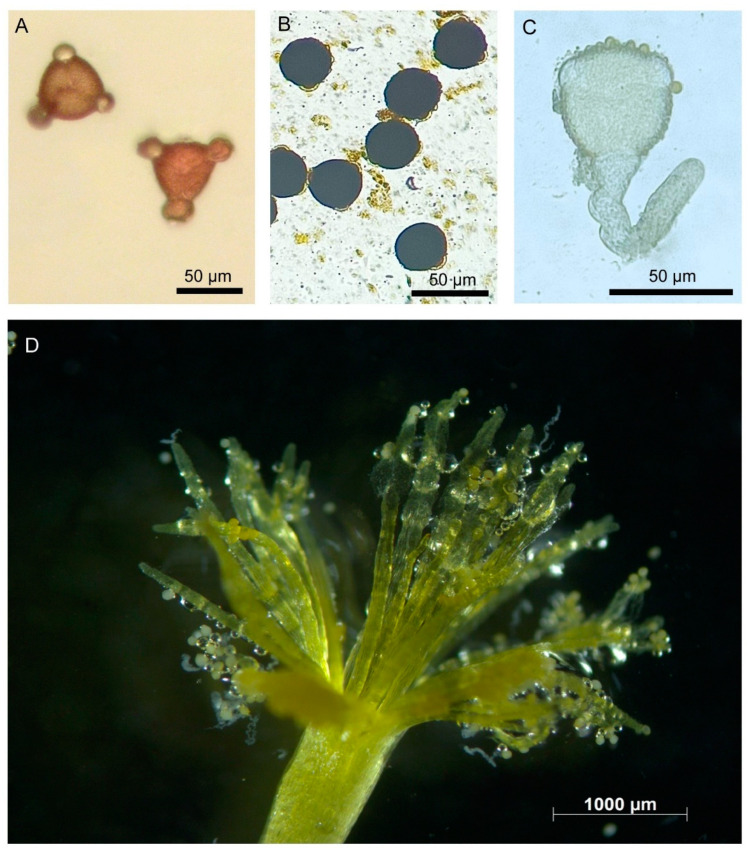
(**A**) Pollens stained with TTC, (**B**) pollens stained with IKI, (**C**) a germinated pollen, and (**D**) stigma showing receptivity.

Anther dehiscence started before the completion of the flower opening. Anther dehisced by a longitudinal slit on the theca in each locule. At opening time, pollen viability was 77.56 ± 6.08% for the TTC test and 84.49 ± 6.33% for the IKI test (Table S2). Pollen germinability was 72.56 ± 6.17%. Pollen viability and germinability did not vary according to season. At the time of anther dehiscence, the stigma remained non-receptive, and it became receptive during the completion of the opening of a flower. Stigma remained receptive throughout the anthesis time (i.e., in opened flowers). Peak receptivity was during 8.00–10.00 h. The duration of stigma receptivity varied season-wise (χ^2^ = 84.32, df = 5, *p* < 0.001), with higher during winter (9.44 ± 0.51 h) and lower in summer (5.88 ± 0.34) (Figure 5 showing viable pollens and receptive stigma). The duration of stigma receptivity negatively correlated with temperature, relative humidity, and day length (temperature: Spearman’s rho = –0.67; RH: Spearman’s rho = –0.47; day length: Spearman’s rho = –0.67; *p* < 0.001, n = 96) and positively correlated with flower longevity (Spearman’s rho = 0.98; *p* < 0.001, n = 96).

### 3.2. Mating System and Reproduction

All five pollination treatments resulted in fruit and seed sets (Table S3). Fruit set was very high (i.e., 100%, excluding damaged flowers). Seed sets in selfing and crossing treatments were almost similar. Therefore, the plant species was fully self-compatible (index of self-compatibility, ISI = 0.02). The seed set in the pollinator exclusion treatment was comparatively lower than supplementary pollination treatments. The plant species showed moderate dependency on pollinators (IDP = 0.34). The value of the coefficient of the pollination deficit was meagre (D = 0.07), indicating there was no pollination limitation of the plant species in an open system.

**Figure 6:**
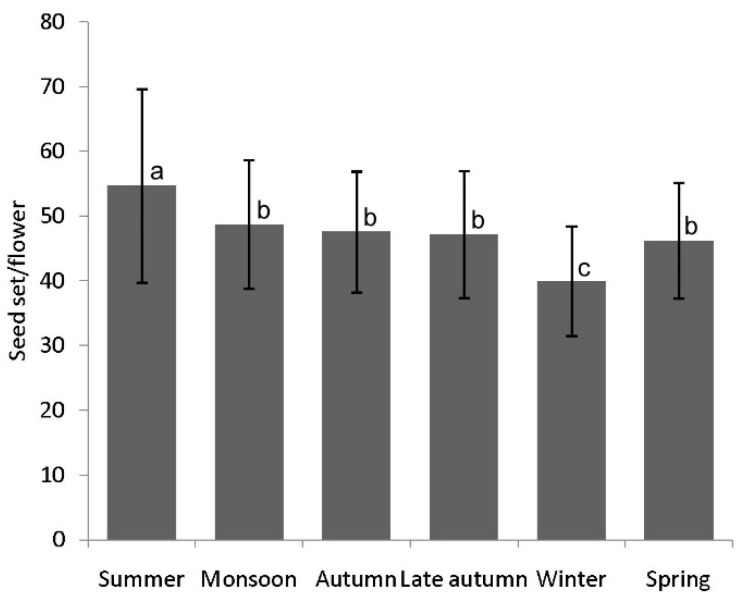
Season-wise seed set (number of seeds) per flower. Values are given in mean ± standard deviation. Different letters indicate significant differences (Dunn’s post hoc test, *p* < 0.05).

The reproductive success (in terms of seed set) largely varied according to season (χ^2^ = 31.14, df = 5, *p* < 0.001). In summer, the plant had a greater seed set (54.72 ± 14.96 seeds/flower) and lower during winter (39.98 ± 8.92 seeds/flower) (Figure 6).

### 3.3. Floral Visitors

**Figure 7:**
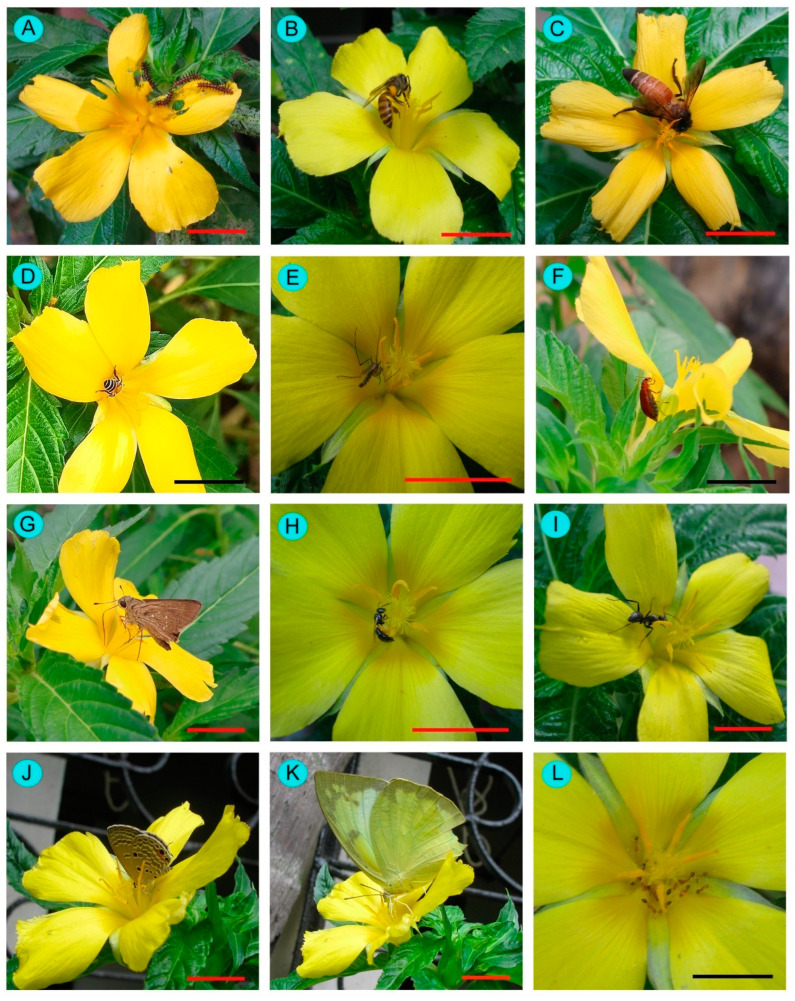
Floral visitors of *Turnera ulmifolia* in West Bengal. (**A**) *Acraea terpsicore*, (**B**) *Apis cerana*, (**C**) *Apis dorsata*, (**D**) *Apis florea*, (**E**) *Armigeres subalbatus*, (**F**) *Aulacophora foveicollis*, (**G**) *Borbo cinnara*, (**H**) *Braunsapis mixta*, (**I**) *Camponotus parius*, (**J**) *Catochrysops panormus*, (**K**) *Catopsilia pumona*, and (**L**) *Crematogastor laestrygon*. Scale bars = 10 mm.

**Figure 8:**
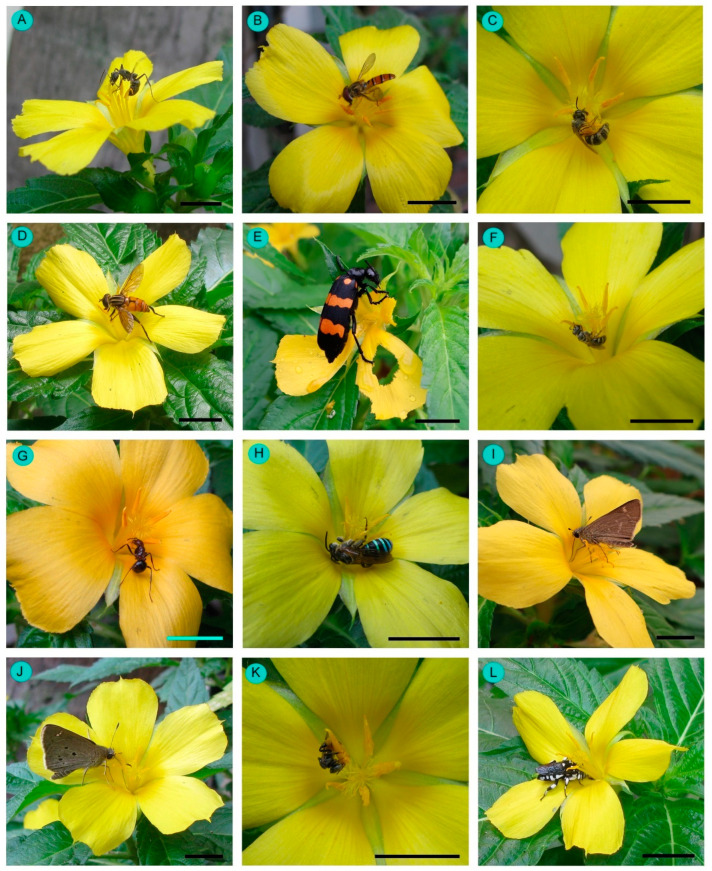
Floral visitors of *Turnera ulmifolia* in West Bengal. (**A**) *Diacamma indicum*, (**B**) *Episyrphus balteatus*, (**C**) *Halictus acrocephalus*, (**D**) *Helophilus peregrinus*, (**E**) *Hycleus phalarantha*, (**F**) *Lasioglossum cavernifrons*, (**G**) *Myrmicaria brunnea*, (**H**) *Nomia* (*Curvinomia*) *strigata*, (**I**) *Pelopidas mathias*, (**J**) *Saustas gremias*, (**K**) *Tetragonula iridipennis*, and (**L**) *Thyreus nitidulus*. Scale bars = 10 mm.

A total of 27 insect species were documented as floral visitors of *Turnera ulmifolia* in West Bengal, India (Table 2, Figure 7 and Figure 8). The most represented insect orders were Hymenoptera (15 species), followed by Lepidoptera (7 species), Diptera (3 species), and Coleoptera (2 species). Among the hymenopteran members, most belong to Apidae (8 species), followed by Formicidae (4 species of ants) and Halictidae (3 species). The butterflies belong to the insect families Hesperiidae (3 species), Lycaenidae (1 species), Nymphalidae (1 species), and Pieridae (2 species).

Visitors’ abundance, richness, and diversity varied seasonally (Table 3). The highest abundance, richness, and diversity were recorded during summer (abundance: 4.89 ± 2.97 visitors/5 min/plant; richness, D = 1.13 ± 0.66; the Shannon–Weaver diversity index, *H′* = 0.87 ± 0.52) and the lowest in winter (abundance: 1.94 ± 1.71 visitors/5 min/plant; richness, D = 0.45 ± 0.60; Diversity *H′* = 0.32 ± 0.43). The visitor abundance positively correlated with flowering intensity (Spearman’s rho = 0.71, *p* < 0.001, n = 96), temperature (Spearman’s rho = 0.60, *p* < 0.001, n = 96), and light (Spearman’s rho = 0.36, *p* < 0.001, n = 96) while negatively correlated with rainfall (Spearman’s rho = −0.42, *p* < 0.001, n = 96). Visitor traits (abundance, richness, and diversity) significantly varied daytime-wise (Table S4). The abundance, richness, and diversity remained higher during 8.00–10.00 h and lower at 12.00–14.00 h.

The abundant flower-visiting species were *Amegilla zonata* (abundance = 0.22 ± 0.58 individuals/plant/5 min; relative abundance = 5.87%), *Borbo cinnara* (abundance = 0.39 ± 0.82 individuals/plant/5 min; relative abundance = 10.61%), *Halictus acrocephalus* (abundance = 0.48 ± 0.94 individuals/plant/5 min; relative abundance = 13.08%), *Lasioglossum cavernifrons* (abundance = 0.28 ± 0.66 individuals/plant/5 min; relative abundance = 7.50%), *Nomia* (*Curvinomia*) *strigata* (abundance = 0.43 ± 0.83 individuals/plant/5 min; relative abundance = 11.60%), and *Tetragonula iridipennis* (abundance = 0.79 ± 1.28 individuals/plant/5 min; relative abundance = 21.57%) (Table 2).

**Figure 9:**
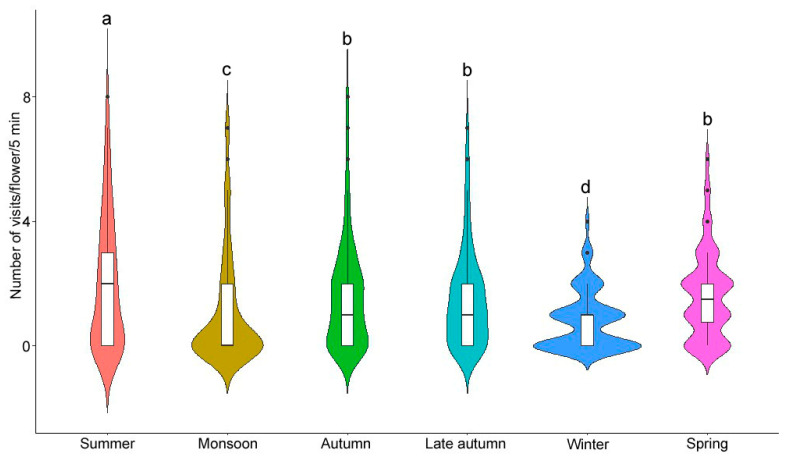
Violin plot showing the season-wise number of visits received by a flower. Different letters indicate significant differences (Dunn’s post hoc test, *p* < 0.05).

The number of visits received by a flower per unit of time differed among the seasons (χ^2^ = 46.98, df = 5, *p* < 0.001). Comparatively, a higher number of visits were received during summer (2.02 ± 2.12 visits/flower/5 min) and a lower number of visits during winter (0.81 ± 0.95 visits/flower/5 min) (Figure 9). Daytime-wise, the maximum number of visits received was during 8–10 h, and the lowest number was during the afternoon (Table S5).

### 3.4. Pollination Strategies

**Figure 10:**
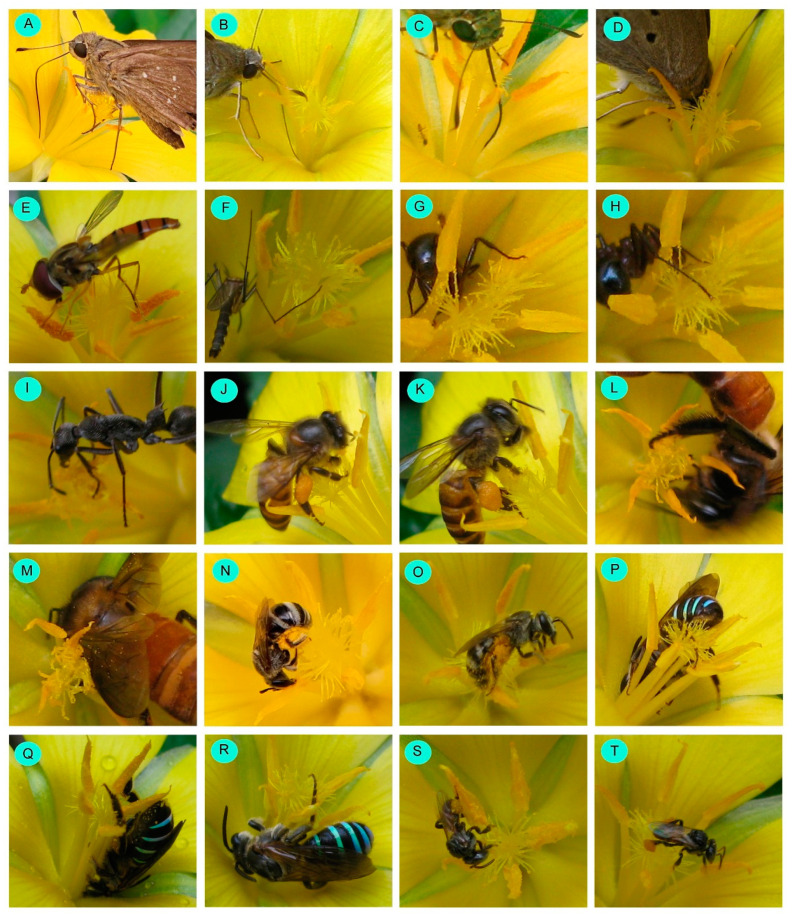
Pollinating strategies of floral visitors. (**A**–**D**) Butterflies, legs, antennae, proboscis, and wings, respectively; (**E**) fly touching through legs and ventral side of thorax and abdomen; (**F**) mosquito touching through legs; (**G**–**I**) ants touching through legs and antennae; (**J**–**M**) honeybees touching through the ventral side of thorax and abdomen, legs, corbicular pollen loads, and wings; (**N**,**O**) *Halictus acrocephalus* touching through the ventral side of thorax and abdomen, legs, scopal pollen loads; (**P**–**R**) *Nomia* (*Curvinomia*) *strigata* touching through the ventral side of the abdomen and legs and showing pulsatory pollination; (**S**,**T**) stingless bees touching through the ventral side of thorax and abdomen, legs, and corbicular pollen loads.

All flower-visiting species legitimately visited *Turnera ulmifolia* flowers (except the larvae of the tawny castor butterfly (*Acraea terpsichore*) and beetles) (Table 4). Butterflies have transferred pollens on stigma through the ventral side of the thorax and abdomen and also through their delicate body parts, including legs, antennae, proboscis, and wings (Figure 10A–D). Flies provided pollination services through their legs. Mosquitoes also touched stigmatic surfaces through their legs (Figure 10F) and provided pollination services. Sometimes, ants touched anthers and stigmatic lobes through their legs. Honeybees pollinated in a sternotribic manner (through the ventral side of the thorax and abdomen). Their legs also touched the stigmas, and occasionally, corbicular pollen loads touched the stigmatic surface. Rarely was pollination carried out through their wings. Solitary bees, when they collected nectar and pollen, touched stigmas through their legs and the ventral side of the thorax and abdomen. Sometimes, they (especially *Braunsapis mixta*, *Halictus acrocephalus*, *Lasioglossum cavernifrons*) travelled from one anther to another of a flower over the stigmatic surfaces and smeared pollens on stigmas. *Nomia* (*Curvinomia*) *strigata* showed different visitation patterns, including vertical visits with the bee’s ventral side remaining facing the flower’s centre (Figure 10P–Q). In most cases, their abdomens touched the flower anthers. During nectar suction from the flowers, they showed the ‘pulsatory movement’ of their abdomen part. They undulated the anthers through their abdominal oscillation, resulting in pollen release, and provided pollination services (i.e., pulsatory pollination). Stingless bees (*Tetragonula iridipennis*) touched stigmas through their legs during pollen collection from an anther. They also travelled over the stigmatic surfaces to move from one anther to another and provided pollination services (Figure 10T).

Single-visit pollination efficiencies (PE*i*) were higher for *Amegilla zonata* (PE*i* = 0.66), *Tetragonula iridipennis* (PE*i* = 0.62), *Halictus acrocephalus* (PE*i* = 0.52), and *Nomia* (*Curvinomia*) *strigata* (PE*i* = 0.50). Pollinator importance (PI) value was the highest for *Amegilla zonata*, followed by *Nomia* (*Curvinomia*) *strigata*, *Halictus acrocephalus*, *Tetragonula iridipennis,* and *Borbo cinnara* (Table 4). These insect species provided significant pollination services to the plant and were considered effective pollinators for *Turnera ulmifolia*.

## 4. Discussion

Several researchers have classified the flowering patterns of plants differently. For example, Gentry [6] classified the species of Bignoniaceae into five categories (steady state, modified steady state, cornucopia, big bang, and multiple bang); Bawa [53] segregated the flowering patterns of plants into two groups (massive and extended); and Frankie et al. [54] classified them into two groups (seasonal and extended). The studied plant species showed steady-state flowering throughout the year. The steady-state flowering increases the chance of cross-pollination. It also may assure reproductive success against adverse weather conditions. All individuals bloomed synchronously. Due to the flowering synchrony, each plant can exchange genes with most plants, increasing the genetic diversity of the same population [55,56]. Flowering intensity, flower longevity, ovules, and pollen production were influenced by temperature, light, humidity, rainfall, and day length. The sensitivity of floral traits against environmental factors was well documented for many plant species (e.g., Barley [57], cocoa [58], and white yam [59]). These floral traits were favoured during summer–monsoon and declined during winter.

Regarding the maturation of reproductive traits, the plant species was protandrous, which is more common in angiosperms than protogyny [60]. This type of dichogamy is less effective for promoting outcrossing for the species. Here, most flowers may be perceived as self- and non-self-pollens, resulting in both autogamy and xenogamy. Pollen viability and germinability did not differ seasonally. The duration of stigma receptivity varied with seasons and depended on flower longevity. During winter, the longer receptive period may mitigate the low flowering frequency and effort to optimise reproductive success [61]. This feature may be an adaptation that allows biotic pollination under adverse environmental conditions. The duration of female receptivity also depends on whether pollination occurred [62,63]. In addition, long-lived stigmas allow extended cross-pollination opportunities for the plant species.

Fruit and seed sets resulted in bagged flowers, indicating that the plant species spontaneously auto-pollinated. This strategy gives the plant extra assurance of reproductive success in hazardous environments with limited pollinator activity. Seed sets in selfing remained almost equal to the output of crossing treatments, implying the self-compatibility of the plant species. Barrett and Shore [64] worked out the breeding systems of different varieties of *Turnera ulmifolia* and reported the phenomenon of self-compatibility. Self-fertilisation (the pollination referred to as ‘selfing’) can be advantageous in the short term for several reasons. Firstly, plants that self-fertilise gain a 50% transmission advantage over outcrossing plants, as they can both self-fertilise their own ovules and contribute outcross pollen simultaneously [65,66]. Secondly, selfing is beneficial in environments where pollinators or potential mates are scarce, providing reproductive assurance [67,68]. Additionally, selfing may enhance colonisation ability [69]. However, a key factor opposing the shift to selfing is inbreeding depression, which refers to the reduced fitness of inbred offspring compared to outcrossed progeny [70]. The seed set in the pollinator exclusion treatment was relatively lower than in the supplementary pollination treatments, indicating that the plant species relied moderately on biotic pollinators. Other species of *Turnera* also depend on pollinators for reproduction (e.g., *Turnera subuata* [71]). The value of the coefficient of pollination deficit was meagre (D = 0.07), indicating there was no pollination limitation of the plant species in an open system. Successful pollination of plant species depends on their flower characteristics, environmental factors, and pollinator activity [48,72].

The reproductive success of the plant species largely varied seasonally, with higher during summer and lower during winter. The seed set depends mainly on successful pollination, linked to flowering intensity, weather conditions, and pollinator activity [73,74]. In summer, some floral traits (e.g., flowering intensity, pollen and ovule production, etc.) remained higher than in winter. More ovule production was also linked to a higher seed set. The relationship between ovule production and seed set was established by many researchers (e.g., Strelin and Aizen [75]; Cucinotta et al. [76]). In addition, high flowering intensity may attract more pollinators, which results in higher reproductive success. The seed sets in four seasons (e.g., monsoon, autumn, late autumn, and spring) did not significantly differ, though environmental factors varied. In the monsoon season, flowering intensity, pollen, and ovule production were higher than in the other three seasons. In contrast, visitor abundance was lower in the monsoon due to adverse atmospheric conditions that may have constrained plant reproduction.

Here, we first recorded detailed floral visitors of *Turnera ulmifolia*. Many insect species (here, 27) visited flowers. A similar number of insect species was also reported for another species, *Turnera subulata*, in NE Brazil [71]. Meanwhile, fewer flower-visiting species were recorded for *Turnera subulata* in Bahia, Brazil [77]. The visitor spectrum depends on plant species and varies across geographical regions [41]. Visitor abundance, richness, and diversity remained higher during summer and lower during monsoon and winter. Environmental variables highly influenced visitor traits and positively influenced flowering intensity. Many researchers (e.g., McCall and Primack [78]; Goodwin et al. [32]) also revealed that flowering patterns and environmental factors are the most important variables influencing insect visitation rates. In addition to atmospheric factors, plant–visitor interactions are also influenced by factors such as habitat fragmentation [79] and pesticide use [80]. The most abundant visitors for *Turnera ulmifolia* in West Bengal were *Borbo cinnara*, *Halictus acrocephalus*, *Nomia* (*Curvinomia*) *strigata*, and *Tetragonula iridipennis*. The visitor abundance largely depends on the plant’s floral architecture, floral resources, and surrounding vegetation that deter the habitat and foods for native pollinators [81,82].

Diverse insect groups (e.g., flies, butterflies, ants, honeybees, solitary bees, and stingless bees) pollinated the plant species. Mosquitoes also legitimately visited the flowers. However, their abundance as visitors and visitation rate were very low and played a minor role in plant reproduction. Ant pollination (i.e., myrmecophily) is not very common in angiosperms, only reported for a few plant species (e.g., Vega et al. [83]; Dutton and Frederickson [84]). Here, ants were attracted by floral tissues, flower nectar, and extrafloral nectar [83], and reproductive success was enhanced by pollinating and helping in seed dispersal [85]. Many solitary bees (e.g., *Amegilla zonata*, *Halictus acrocephalus*, *Lasioglossum cavernifrons*, *Nomia* (*Curvinomia*) *strigata*, etc.) provided pollination services to the plant species. The solitary bee-mediated pollination was also recorded for other species, e.g., *Turnera subulata* [71,77]. Most visitors pollinated *Turnera ulmifolia* flowers sternotribically through the ventral surface of the thorax and abdomen. The pollination mode for a plant species largely depends on pollinator species and flower architecture [86]. Funnel-shaped flowers with exposed anthers may serve as sternotribic flowers for most visitors. The visitors transferred pollen to the stigmatic surface through their thorax and abdomen as well as delicate parts such as the legs, antennae, proboscis, and wings. The centrally placed stigmatic branches provided landing substrates for visitors, and most pollinators travelled from one anther to another over the stigmas and provided pollination services. Additionally, *Nomia* (*Curvinomia*) *strigata* performed another pollination mode, i.e., pulsatory pollination. This phenomenon was the first time we reported it for a pollinator species. In this case, the ventral surface of the abdomen was touched by anthers, and the oscillation of the abdomen caused the release of pollens from the anther and then to be deposited on stigmas. Though many insect species provided pollination services to the plant species, their efficiency (considering pollination services) varied. The pollination efficiency of pollinators depends on their abundance, foraging activity, resource-collecting behaviour, and flower traits [31]. Here, we estimated the pollinator importance (PI) of visitor species by considering relative abundance, flower visitation rate, and single-visit pollination efficiency. Based on the PI value, effective pollinators were *Amegilla zonata*, *Borbo cinnara*, *Halictus acrocephalus*, *Nomia* (*Curvinomia*) *strigata,* and *Tetragonula iridipennis*.

## 5. Conclusions

The study depicted the influences of atmospheric factors (e.g., temperature, light intensity, humidity, and rainfall) and photoperiod on floral biology, plant–pollinator interactions, and the reproductive success of *Turnera ulmifolia*. Temperature, light, humidity, and day length positively impacted flowering intensity, pollen, and ovule production. Temperature, humidity, and day length negatively influenced the flower longevity. Consequently, higher flowering intensity, pollen, and ovule production were recorded during the hot and humid summer and monsoon compared to the cold winter. Floral visitor abundance, richness, and diversity positively correlated with flowering intensity and were negatively influenced by rainfall. Therefore, visitor traits remained higher during summer and lower during monsoon and winter. Diverse insect groups pollinated the plant species, including ants, bees, butterflies, and flies. Effective pollinators were *Amegilla zonata*, *Borbo cinnara*, *Halictus acrocephalus*, *Nomia* (*Curvinomia*) *strigata,* and *Tetragonula iridipennis*. Most pollinators showed a sternotribic pollination mode, carried out through the ventral surface of the thorax and abdomen and through their delicate parts like legs, antennae, proboscis, and wings. *Nomia* (*Curvinomia*) *strigata* showed a unique mode of pollination, i.e., pulsatory pollination. Considering reproductive success, higher reproductive fitness was revealed during the hot summer, while comparatively lower reproductive success was revealed during the cold winter. Therefore, it can be concluded that the floral biology, plant-pollinator interactions, and reproductive success of *Turnera ulmifolia* varied seasonally, with significant influences from atmospheric factors and photoperiod.

## Figures and Tables

**Table 1:** Season-wise floral traits of *Turnera ulmifolia* in West Bengal.

Season	Flowering Intensity	Flower Opening Time	Flower Closing Time	Flower Longevity	Pollen/Flower	Ovule/Flower
Summer	8.96 ^a^ ± 5.30	5.00–6.00 h	10.00–11.00 h	4.88 ^b^ ± 0.34	14,075 ^a^ ± 323.93	60.96 ^a^ ± 11.07
Monsoon	8.48 ^a^ ± 5.31	5.00–6.00 h	10.00–11.00 h	5.00 ^b^ ± 0	14,133 ^a^ ± 370.78	61.60 ^a^ ± 13.69
Autumn	8.17 ^a^ ± 5.17	5.00–6.00 h	11.00–12.00 h	6.31 ^ab^ ± 0.48	13,685.42 ^ab^ ± 179.13	52.54 ^ab^ ± 6.26
Late autumn	7.48 ^ab^ ± 4.92	6.00–7.00 h	13.00–14.00 h	7.38 ^a^ ± 0.50	13,358.76 ^ab^ ± 292.26	51.79 ^ab^ ± 7.56
Winter	2.31 ^b^ ± 1.67	8.00–9.00 h	15.00–17.00 h	8.25 ^a^ ± 0.45	11,389.62 ^b^ ± 497.49	44.42 ^b^ ± 7.42
Spring	7.54 ^ab^ ± 4.95	7.00–8.00 h	12.00–13.00 h	5.25 ^b^ ± 0.45	13,918.31 ^a^ ± 294.78	51.46 ^ab^ ± 8.82
Throughout year	7.16 ^ab^ ± 5.20	5.00–9.00 h	10.00–17.00	6.18 ^ab^ ± 1.34	13,426.68 ^ab^ ± 1008.01	53.80 ^ab^ ± 11.12
Statistical analysis	χ^2^ = 52.78, df = 5, *p* < 0.001	-	-	χ^2^ = 263.52, df = 5, *p* < 0.001	χ^2^ = 67.96, df = 5, *p* < 0.001	χ^2^ = 79.64, df = 5, *p* < 0.001

Values are given in mean ± standard deviation. Different superscript letters within a column indicate significant differences (Dunn’s post hoc test at 0.05% level).

**Table 2:** Flower visitors of *Turnera ulmifolia* in West Bengal, India.

Floral Visitors	Abundance	Relative Abundance (%)	Flower Visitation Rate	Flower Handling Time	Floral Resources
▪ Coleoptera					
*Aulacophora foveicollis*	<0.01	0.21	-	-	FT
*Hycleus phalarantha*	<0.01	0.14	-	-	FT
▪ Diptera					
*Armigeres subalbatus*	0.02 ± 0.15	0.64	-	-	N
*Episyrphus balteatus*	0.05 ± 0.22	1.41	2.95 ± 1.00	13.12 ± 14.28	P
*Helophilus peregrinus*	0.04 ± 0.20	1.13	-	-	P
▪ Hymenoptera					
*Amegilla zonata*	0.22 ± 0.58	5.87	4.30 ± 1.56	6.27 ± 3.19	N + P
*Apis cerana*	0.06 ± 0.24	1.56	3.10 ± 0.91	13.50 ± 13.66	N + P
*Apis dorsata*	0.05 ± 0.22	1.34	3.70 ± 1.03	10.52 ± 5.23	N + P
*Apis florea*	0.03 ± 0.17	0.78	3.35 ± 0.93	12.26 ± 10.31	N + P
*Braunsapis mixta*	0.05 ± 0.24	1.34	1.16 ± 0.30	46.24 ± 16.29	N + P
*Camponotus parius*	0.14 ± 0.47	3.68	-	-	FT, N + P
*Ceratina compacta*	0.05 ± 0.22	1.27	-	-	N + P
*Crematogaster laestrygon*	0.14 ± 0.53	3.82	-	-	FT, N + P
*Diacamma indicum*	0.05 ± 0.26	1.41	-	-	FT, N + P
*Halictus acrocephalus*	0.48 ± 0.94	13.08	1.46 ± 0.28	39.43 ± 14.25	N + P
*Lasioglossum cavernifrons*	0.28 ± 0.66	7.50	1.29 ± 0.32	42.18 ± 14.62	N + P
*Myrmicaria brunnea*	0.13 ± 0.47	3.61	-	-	FT, N + P
*Nomia strigata*	0.43 ± 0.83	11.60	1.88 ± 0.43	36.24 ± 12.35	N + P
*Tetragonula iridipennis*	0.79 ± 1.28	21.57	0.71 ± 0.28	57.18 ± 26.44	N + P
*Thyreus nitidulus*	0.03 ± 0.16	0.71	3.75 ± 1.12	8.37 ± 3.71	N
▪ Lepidoptera					
*Acraea terpsicore*	0.05 ± 0.28	1.41	-	-	FT
*Borbo cinnara*	0.39 ± 0.82	10.61	4.20 ± 1.47	13.47 ± 11.76	N
*Catochrysops panormus*	0.03 ± 0.17	0.78	1.40 ± 1.12	21.30 ± 18.29	N
*Catopsilia pomona*	0.02 ± 0.14	0.64	2.15 ± 1.30	8.74 ± 7.56	N
*Eurema hecabe*	0.02 ± 0.17	0.78	1.72 ± 1.43	17.61 ± 14.82	N
*Pelopidas mathias*	0.09 ± 0.34	2.40	4.05 ± 1.39	14.93 ± 12.25	N
*Suastus gremius*	0.03 ± 0.16	0.71	2.30 ± 2.11	18.46 ± 15.03	N

FT: floral tissue, N: nectar, P: pollen. Values are given as mean ± standard deviation.

**Table 3:** Abundance (number of visitors/individual/5 min), richness (Margalef’s index D), and diversity (index of Shannon–Weaver, *H*’) of floral visitors on *Turnera ulmifolia* in West Bengal.

Season	Abundance	Richness	Diversity
Summer	4.89 ^a^ ± 2.97	1.13 ^a^ ± 0.66	0.87 ^a^ ± 0.52
Monsoon	3.23 ^b^ ± 2.78	0.74 ^b^ ± 0.70	0.56 ^b^ ± 0.53
Autumn	4.06 ^ab^ ± 2.93	0.91 ^ab^ ± 0.64	0.71 ^ab^ ± 0.52
Late autumn	3.94 ^ab^ ± 2.36	0.96 ^ab^ ± 0.62	0.72 ^ab^ ± 0.48
Winter	1.94 ^c^ ± 1.71	0.45 ^c^ ± 0.60	0.32 ^c^ ± 0.43
Spring	4.03 ^ab^ ± 2.54	0.94 ^ab^ ± 0.66	0.73 ^ab^ ± 0.51
Throughout year	3.68 ± 2.54	0.85 ± 0.68	0.65 ± 0.52
Statistical analysis	χ^2^ = 44.10, df = 5, *p* < 0.001	χ^2^ = 36.42, df = 5, *p* < 0.001	χ^2^ = 42.49, df = 5, *p* < 0.001

Values are given as mean ± standard deviation. Different superscript letters within a column indicate significant differences (Dunn’s post hoc test, *p* < 0.05).

**Table 4:** Pollinating strategies of flower visitors on *Turnera ulmifolia*.

Visitors	Visitation Type	Mode of Pollination	PE*i*	PI
Ants	**	S; ventral side of thorax and abdomen, legs	-	-
Beetles	IV	-	-	-
Butterflies				
*Acraea terpsicore*	IV	-	-	-
*Borbo cinnara*	***	S; ventral side of thorax and abdomen, legs, antennae, proboscis, wings	0.21	9.36
*Catochrysops panormus*	***	S; ventral side of thorax and abdomen, legs, antennae, proboscis, wings	-	-
*Catopsilia pomona*	***	S; ventral side of thorax and abdomen, legs, antennae, proboscis, wings	-	-
*Eurema hecabe*	***	S; ventral side of thorax and abdomen, legs, antennae, proboscis, wings	-	-
*Pelopidas mathias*	***	S; ventral side of thorax and abdomen, legs, antennae, proboscis, wings	0.21	2.04
*Suastus gremius*	***	S; ventral side of thorax and abdomen, legs, antennae, proboscis, wings	-	-
Flies	**	S; ventral side of thorax and abdomen, legs	-	-
Mosquitoes	*	Legs	-	-
Honeybees	***	S; ventral side of thorax and abdomen, legs, wings, corbicular pollen loads	-	-
Solitary bees				
*Amegilla zonata*	***	S; ventral side of thorax and abdomen, legs,	0.66	16.66
*Braunsapis mixta*	***	S; ventral side of thorax and abdomen, legs,	-	-
*Ceratina compacta*	***	S; ventral side of thorax and abdomen, legs,	-	-
*Halictus acrocephalus*	***	S; ventral side of thorax and abdomen, legs, scopal pollen loads	0.52	9.93
*Lasioglossum cavernifrons*	***	S; ventral side of thorax and abdomen, legs,	0.47	4.55
*Nomia* (*Curvinomia*) *strigata*	***	S, pulsatory pollination; ventral side of thorax and abdomen, legs,	0.50	10.90
*Thyreus nitidulus*	**	S; ventral side of thorax and abdomen, legs,	-	-
Stingless bees	***	S; ventral side of thorax and abdomen, legs, corbicular pollen loads	0.62	9.50

PE*i*: single-visit pollination efficiency index, PI: pollinator importance, S: sternotribic, *: rarely legitimate visit, **: frequently legitimate visit, ***: mostly legitimate visit, IV: illegitimate visit.

## Data Availability

The data presented in this study are available upon request from the corresponding author.

## Supplementary Materials

The following supporting information can be downloaded at: https://www.mdpi.com/article/10.3390/biology14010100/s1, Table S1: The methodologies for the microscopic study of flower parts and pollen grains; Table S2: Pollen viability, germinability, and stigma receptivity (duration in hours) of *Turnera ulmifolia*; Table S3: Reproductive success (seed set per flower) of *Turnera ulmifolia* in different pollination treatments; Table S4: Daytime-wise, visitor traits (abundance, richness, and diversity) of *Turnera ulmifolia* in West Bengal; Table S5: Daytime-wise, the number of visits received a flower (number of visits/flower/5 min) of *Turnera ulmifolia* in West Bengal.
