# Supplementary material for: Impact of Seasonal Atmospheric Factors and Photoperiod on Floral Biology, Plant–Pollinator Interactions, and Plant Reproduction on Turnera ulmifolia L. (Passifloraceae)
Source: Biology (Basel). 2025 Jan 19;14(1):100. doi: 10.3390/biology14010100 (PMC11760852; doi:10.3390/biology14010100)
Supplement: Supplementary file 1 [file biology-14-00100-s001.zip › Table S1.pdf]

Table S1. The methodologies for the microscopic study of flower parts and pollen grains.

Phase	Different steps
Flower parts (e.g., sepal, stigma, and ovary) preparation for scanning electron microscope (SEM)	1. A small piece of the flower parts (e.g., sepal, stigma, and ovary) were taken in vials.<br>2. Flower parts were fixed in a 3% glutaraldehyde solution.<br>3. After discarding the glutaraldehyde solution, the samples were gradually dehydrated using ethanol at increasing concentrations (50%, 70%, and 100%).<br>4. Then, samples were air-dried.
SEM study of flower parts	1. The flower samples were affixed to metal stubs using sticky carbon tape to enhance conductivity.<br>2. To mitigate charge buildup on the specimen surface, a 10 nm layer of gold nanoparticles was applied as a conductive coating using the Q150R ES sputter coater (Quorum Technologies, UK).<br>3. The samples were examined with a Merlin emission gun scanning electron microscope (FEG-SEM) from Zeiss, Gemini, India. Surface images of the pollen samples were captured under high vacuum conditions at magnifications ranging from 1 KX to 40 KX. Imaging was performed using a secondary electron detector with an acceleration voltage of 5.0 kV.
Pollen sample preparation for light microscope	1. Pollen grains were collected from flower anthers.<br>2. Pollen grains were stored in a vial with 70% ethanol.
Light microscopic study of pollen grains	1. Pollen solution was centrifuged at 4000 rpm for 5 min.<br>2. After decanting the supernatant, the resulting pollen sediment was taken on a glass slide with glycerine jelly.<br>3. The jelly-containing pollen was warmed to melt it and covered with a coverslip.<br>4. We sealed the coverslip by using nail polish.<br>5. Then, we observed the pollens using a light microscope (Primo Star, Zeiss) and took pollen microphotographs.
Pollen sample preparation for SEM study	3. Pollen grains were collected from flower anthers.<br>4. Pollen grains were fixed in a 3% glutaraldehyde solution.<br>5. Then, discarded glutaraldehyde by centrifugation.<br>6. Then, pollen grains were dehydrated gradually using ethanol with increasing ethanol concentrations.<br>7. After centrifugation, pollen grains were air-dried.
SEM study of pollen grains	4. The pollen samples were affixed to metal stubs using sticky carbon tape to enhance conductivity.<br>5. Then, we followed a similar methodology to the SEM study of flower parts.
