# Supplementary material for: Impact of Seasonal Atmospheric Factors and Photoperiod on Floral Biology, Plant–Pollinator Interactions, and Plant Reproduction on Turnera ulmifolia L. (Passifloraceae)
Source: Biology (Basel). 2025 Jan 19;14(1):100. doi: 10.3390/biology14010100 (PMC11760852; doi:10.3390/biology14010100)
Supplement: Supplementary file 1 [file biology-14-00100-s001.zip › Table S2.pdf]

Table S2. Pollen viability, germinability, and stigma receptivity (duration in hours) of *Turnera ulmifolia*.

**Season**	**Pollen viability (%)**		**Pollen germinability (%)**	**Stigma receptivity (h)**
	TTC	IKI		
Summer	77.92 ± 6.31	84.88 ± 6.26	73.34 ± 6.29	5.88 ± 0.34
Monsoon	78.21 ± 6.08	85.45 ± 6.27	74.17 ± 6.15	6.12 ± 0.34
Autumn	78.04 ± 6.33	85.12 ± 6.31	73.81 ± 6.43	7.38 ± 0.50
Late autumn	77.76 ± 6.06	84.57 ± 6.62	72.62 ± 5.92	8.44 ± 0.51
Winter	75.87 ± 6.28	82.62 ± 6.46	70.28 ± 5.94	9.44 ± 0.51
Spring	77.56 ± 6.07	84.32 ± 6.67	72.40 ± 6.15	6.44 ± 0.51
Throughout year	77.56 ± 6.08	84.49 ± 6.33	72.77 ± 6.14	7.24 ± 1.37
Statistical analysis	χ^2 = 1.94, df = 5, p = 0.86	χ^2 = 2.20, df = 5, p = 0.82	χ^2 = 1.87, df = 5, p = 0.88	χ^2 = 84.32, df = 5, p < 0.001
