# Supplementary material for: Impact of Seasonal Atmospheric Factors and Photoperiod on Floral Biology, Plant–Pollinator Interactions, and Plant Reproduction on Turnera ulmifolia L. (Passifloraceae)
Source: Biology (Basel). 2025 Jan 19;14(1):100. doi: 10.3390/biology14010100 (PMC11760852; doi:10.3390/biology14010100)
Supplement: Supplementary file 1 [file biology-14-00100-s001.zip › Table S3.pdf]

Table S3. Reproductive success (seed set per flower) of *Turnera ulmifolia* in different pollination treatments.

Pollination treatment	Fruit set (%)	Seed set per flower
Open-pollination	100	54.72 ± 14.96
Spontaneous auto pollination	100	38.95 ± 7.11
Manual selfing	100	56.78 ± 10.77
Manual crossing	100	57.72 ± 11.49
Supplementary pollination	100	58.88 ± 12.65
