# Supplementary material for: Impact of Seasonal Atmospheric Factors and Photoperiod on Floral Biology, Plant–Pollinator Interactions, and Plant Reproduction on Turnera ulmifolia L. (Passifloraceae)
Source: Biology (Basel). 2025 Jan 19;14(1):100. doi: 10.3390/biology14010100 (PMC11760852; doi:10.3390/biology14010100)
Supplement: Supplementary file 1 [file biology-14-00100-s001.zip › Table S4.pdf]

**Table S4.** Daytime-wise, visitor traits (abundance, richness, and diversity) of *Turnera ulmifolia* in West Bengal.

| Visitor traits | Summer | Monsoon | Autumn | Late autumn | Winter | Spring |
|---|---|---|---|---|---|---|
| Abundance | | | | | | |
| 6.00-8.00 h | $4.50^{b} \pm 1.83$ | $2.19^{b} \pm 1.52$ | $3.00^{bc} \pm 1.90$ | $2.81^{c} \pm 1.33$ | $0.62^{c} \pm 0.72$ | $2.75^{c} \pm 1.24$ |
| 8.00-10.00 h | $8.50^{a} \pm 1.67$ | $5.75^{a} \pm 3.15$ | $7.12^{a} \pm 2.63$ | $6.81^{a} \pm 1.33$ | $4.00^{a} \pm 1.21$ | $7.25^{a} \pm 1.39$ |
| 10.00-12.00 h | $5.25^{b} \pm 1.48$ | $4.19^{ab} \pm 2.04$ | $4.94^{b} \pm 1.98$ | $4.75^{b} \pm 1.00$ | $2.62^{b} \pm 0.96$ | $4.88^{b} \pm 0.81$ |
| 12.00-14.00 h | $1.31^{c} \pm 0.95$ | $0.81^{c} \pm 0.91$ | $1.19^{c} \pm 0.98$ | $1.38^{d} \pm 1.02$ | $0.50^{c} \pm 0.52$ | $1.25^{d} \pm 1.06$ |
| Richness | | | | | | |
| 6.00-8.00 h | $1.18^{b} \pm 0.48$ | $0.68^{b} \pm 0.68$ | $0.78^{bc} \pm 0.60$ | $0.79^{b} \pm 0.59$ | $0.09^{c} \pm 0.36$ | $0.70^{b} \pm 0.60$ |
| 8.00-10.00 h | $1.65^{a} \pm 0.27$ | $1.14^{a} \pm 0.61$ | $1.39^{a} \pm 0.34$ | $1.37^{a} \pm 0.25$ | $1.02^{a} \pm 0.51$ | $1.38^{a} \pm 0.32$ |
| 10.00-12.00 h | $1.21^{b} \pm 0.51$ | $0.96^{ab} \pm 0.64$ | $1.05^{ab} \pm 0.54$ | $1.24^{a} \pm 0.33$ | $0.68^{b} \pm 0.61$ | $1.29^{a} \pm 0.42$ |
| 12.00-14.00 h | $0.47^{c} \pm 0.73$ | $0.18^{c} \pm 0.49$ | $0.42^{c} \pm 0.65$ | $0.44^{b} \pm 0.70$ | $0^{d}$ | $0.38^{b} \pm 0.61$ |
| Diversity | | | | | | |
| 6.00-8.00 h | $0.89^{b} \pm 0.36$ | $0.44^{b} \pm 0.43$ | $0.59^{c} \pm 0.45$ | $0.51^{b} \pm 0.39$ | $0.04^{c} \pm 0.17$ | $0.48^{bc} \pm 0.41$ |
| 8.00-10.00 h | $1.36^{a} \pm 0.15$ | $0.95^{a} \pm 0.50$ | $1.17^{a} \pm 0.33$ | $1.19^{a} \pm 0.18$ | $0.80^{a} \pm 0.37$ | $1.20^{a} \pm 0.23$ |
| 10.00-12.00 h | $0.98^{b} \pm 0.38$ | $0.74^{ab} \pm 0.49$ | $0.87^{b} \pm 0.43$ | $0.97^{ab} \pm 0.22$ | $0.45^{b} \pm 0.39$ | $1.03^{ab} \pm 0.25$ |
| 12.00-14.00 h | $0.24^{c} \pm 0.38$ | $0.09^{c} \pm 0.24$ | $0.21^{d} \pm 0.32$ | $0.23^{c} \pm 0.37$ | $0^{d}$ | $0.21^{c} \pm 0.32$ |
| Statistical analysis | Abundance: $\chi2$ = 48.79, ***; Richness: $\chi2$ = 21.95, ***; Diversity: $\chi2$ = 36.74, *** | Abundance: $\chi2$ = 28.58, ***; Richness: $\chi2$ = 16.23, **; Diversity: $\chi2$ = 23.99, *** | Abundance: $\chi2$ = 37.36, ***; Richness: $\chi2$ = 16.16, **; Diversity: $\chi2$ = 27.92, *** | Abundance: $\chi2$ = 49.83, ***; Richness: $\chi2$ = 17.46, **; Diversity: $\chi2$ = 36.20, *** | Abundance: $\chi2$ = 45.54, ***; Richness: $\chi2$ = 33.0, ***; Diversity: $\chi2$ = 36.60, *** | Abundance: $\chi2$ = 52.50, ***; Richness: $\chi2$ = 22.87, ***; Diversity: $\chi2$ = 41.24, *** |

Values are given in mean ± standard deviation. Statistical analysis: different superscript letters within a column indicate significant differences (Dunn's post hoc test at 0.05% level); Kruskal-Wallis H test: df = 3, $p<0.001$ (***), $p<0.01$ (**), $p<0.05$ (*).
