# Supplementary material for: Impact of Seasonal Atmospheric Factors and Photoperiod on Floral Biology, Plant–Pollinator Interactions, and Plant Reproduction on Turnera ulmifolia L. (Passifloraceae)
Source: Biology (Basel). 2025 Jan 19;14(1):100. doi: 10.3390/biology14010100 (PMC11760852; doi:10.3390/biology14010100)
Supplement: Supplementary file 1 [file biology-14-00100-s001.zip › Table S5.pdf]

Table S5. Daytime-wise, the number of visits received a flower (number of visits/flower/5 min) of *Turnera ulmifolia* in West Bengal.

Season	Number of visits/flower/5 min				
	6.00-8.00 h	8.00-10.00 h	10.00-12.00 h	12.00-14.00 h	Throughout day
Summer	2.56 ± 1.61	4.50 ± 1.85	1.03 ± 0.78	0	2.02 ± 2.12
Monsoon	0.91 ± 1.65	2.59 ± 2.39	0.94 ± 1.11	0	1.11 ± 1.80
Autumn	1.47 ± 1.52	3.22 ± 2.30	1.19 ± 1.06	0.78 ± 0.87	1.66 ± 1.78
Late autumn	1.34 ± 1.26	3.06 ± 2.03	1.12 ± 0.98	0.72 ± 0.77	1.56 ± 1.61
Winter	0.38 ± 0.66	1.84 ± 1.02	0.59 ± 0.67	0.44 ± 0.56	0.81 ± 0.95
Spring	1.09 ± 0.96	3.03 ± 1.49	1.53 ± 1.11	0.84 ± 0.85	1.62 ± 1.40
